# Relationship Between Elastic, Chemical, and Thermal Properties of SiO_2_ Flint Aggregate

**DOI:** 10.3390/molecules29245898

**Published:** 2024-12-13

**Authors:** Lahcen Khouchaf, Abdelhamid Oufakir

**Affiliations:** 1 IMT Nord Europe, Institut Mines-Telecom, University Lille, Centre for Materials and Processes, F-59000 Lille, France; 2 Department of Chemistry, Faculty of Science, Cadi Ayyad University, Marrakech 40000, Morocco; oufkir.emi@gmail.com

**Keywords:** natural SiO_2_ aggregate, Williamson–Hall, elastic properties, microstrain, thermal treatment, microstructure, silanol SiOH, circular economy

## Abstract

Understanding the relationship between elastic, chemical, and thermal properties is essential for the prevention of the behavior of SiO_2_ flint aggregates during their application. In fact, the elastic properties of silica depend on chemical and heat treatment. In order to identify the crystallite sizes for natural SiO_2_ before and after chemical treatment samples, Williamson–Hall plots and Scherer’s formulas are used. The silica nanofibers obtained and their microstructure changes under thermal and chemical treatment are characterized using different techniques (XRD, VP-SEM, TEM, FTIR, TDA, and TGA). Both the strains (ε) and the crystallite sizes (DW–H) are obtained from the slope and from the βcosθ-intercept of a graph, respectively. The crystalline quality is improved upon heating, as shown by the decrease in the FWHM of the SiO_2_(101) peaks, which is confirmed by Fourier transform infrared spectroscopy (FTIR). The microstrain estimated at 1.50 × 10^−4^ units for natural SiO_2_ is smaller than that for SiO_2_ after chemical attack which is estimated at 2.01 × 10^−4^ units. Based on the obtained results, SiO_2_ characterized with controlled micromechanical, thermal, and chemical properties may be used as a filler to improve the performance properties of the strength, microstructure, and durability of some composites.

## 1. Introduction

New composites based on fillers have recently attracted more attention as additives in cement and/or polymer materials [1,2], as well as in the automotive and aeronautics industry [3], because they are cheaper, environmentally friendly, and energy-efficient. However, the effects of these materials on the performance of these composites have not been elucidated. We have used different techniques in order to move forward in this direction. We performed various treatments and predominantly obtained the nano-silica morphology of the surface. The pretreatment methods employed allow for obtaining new physicochemical and morphological characteristics of SiO_2_ surfaces. Moreover, the influences of different treatments on the microstructure and durability properties are discussed.

Conventional cement production contributes significantly to carbon dioxide emissions. Its importance in the construction sector requires the development of alternative and environmentally friendly building materials. The use of new composites from natural materials like silica SiO_2_ offers a sustainable alternative to traditional cement aiming to create high-performance, environmentally friendly building materials [1].

In addition, the valorization of natural resources is increasingly in demand because they do not have a negative impact on the environment compared to nanoparticles. Similarly, some solid waste valorizations use advanced thermochemical processes [4].

In this case, their recycling after use is even facilitated and leads to the development of new circular economy models [5]. The microstructure changes are controlled when the aggregate is submitted to different thermal environments and/or interacts with various chemical fillers. Many composites with filler insertion are finding increasingly widespread applications in the industrial and academic research sectors. Indeed, silica compounds are widely used in industries as fillers [6,7].

The determination of the chemical activity of silica depends on the silanol groups (SiOH) which are the main surface reactive sites [8,9]. In addition, a strong relationship between reactivity and the structural order of silica compounds exists [10,11].

In fact, because the reactive property of silica mainly comes from the presence of silanol Si–OH groups on its surface [12,13,14,15], the surface of SiO_2_ compounds with Si–O–Si siloxane groups is much less reactive than the surface with Si-OH. For that, it is necessary to create more Si–OH groups on the surface to increase SiO_2_ reactivity and interaction with the molecules of the matrix.

Upon heating, the trigonal α-quartz will transform into hexagonal β-quartz at 573 °C. The temperature influences the structure by modifying the angles and the lengths of the Si–O–Si bonds [15,16,17,18]. For example, the silanol group concentration decreases at a higher temperature by dehydroxylation processes. In fact, two silanol OH groups are released by forming a water molecule and siloxane bonds according to Reaction (1) [16,17]:

2Si–OH → Si–O–Si + H_2_O (1)

The aim of this work is to understand the relationship between the elastic, chemical, and thermal properties of SiO_2_ flint aggregate in order to facilitate its use as filler in composite materials. Micromechanical characterizations were carried out on states in order to identify the most critical parameters for the selection of SiO_2_ aggregate as filler in different applications. Chemical and micromechanical properties were also measured and discussed in order to assess the durability of the studied aggregate.

## 2. Results

### 2.1. Variable Pressure Scanning Electron Microscopy (VP-SEM)

**Figure 1:**
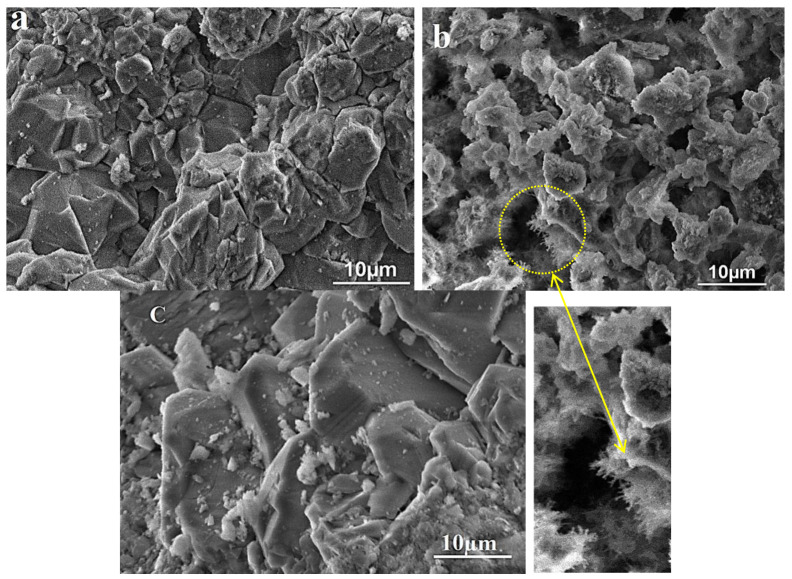
VP-SEM micrographs of the SiO_2_ aggregate: (**a**) natural sample, (**b**) after chemical treatment, and (**c**) after heat treatment.

VP-SEM images before and after reaction are shown in Figure 1. Before reaction, the grains have a relatively smooth surface as a well-defined shape. In addition, some dispersion of the grains (Figure 1a) is observed with different sizes ranging from a few micrometers to a hundred nanometers. Also, the typical angles of the grains characteristic of quartz are observed. However, after chemical treatment a significant morphological change in the grains occurs showing a fibrous surface (Figure 1b). This can be explained by the breaking of Si–O–Si bonds by OH^−^ ions inducing the formation of silanol groups. These results are confirmed below, and they may be considered as a parameter of the functionalization of the SiO_2_ aggregate [18,19]. This functionalization can be used to improve the interface behavior of some composite materials with SiO_2_ compounds as a filler. More significant modifications are produced after heating. As shown in Figure 1c, the grains present a well-defined shape with a smooth surface. These results are in agreement with the XRD results which demonstrate the increase in crystallite size as a function of temperature [20].

### 2.2. High-Resolution Transmission Electron Microscopy

**Figure 2:**
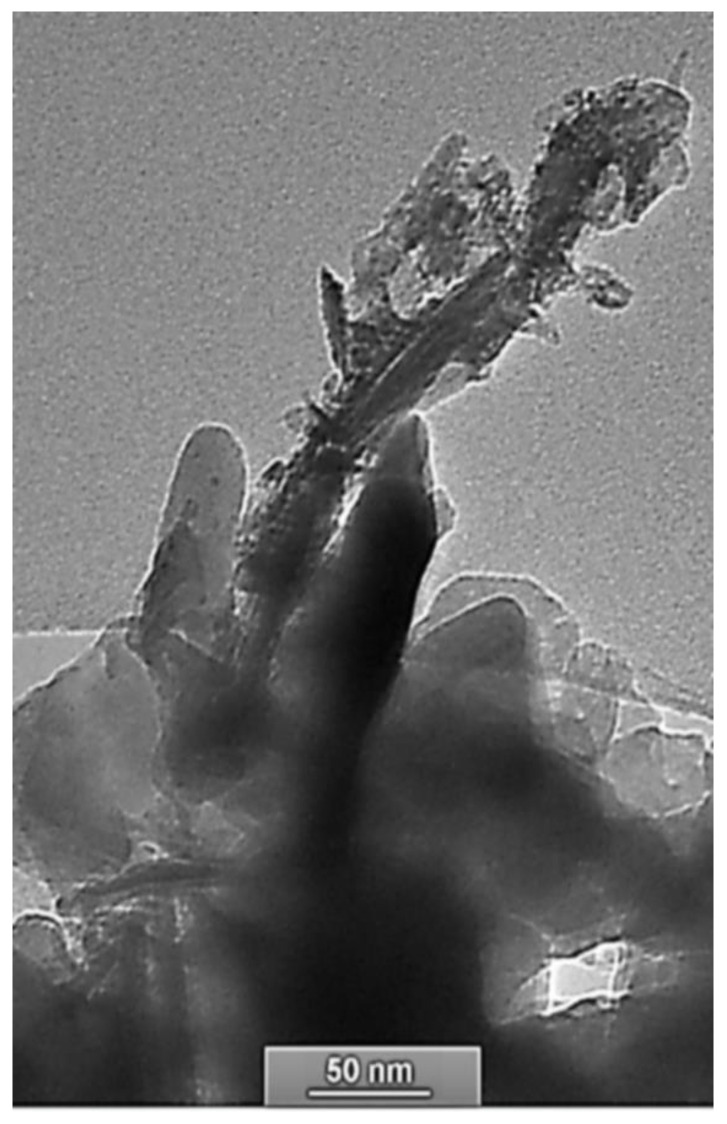
HR-TEM images of SiO_2_ nanofibers on the surface after chemical treatment.

HR-TEM and energy-dispersive X-ray spectroscopy (EDS) are used in order to follow the morphology, the chemical composition, and the nanostructured surface of SiO_2_ aggregate. For SiO_2_ samples after chemical treatment, silica nanofibers with a size of 20–250 nm were formed (Figure 2) on the surface. These nano SiO_2_ fibers adhered to the surface, showing the strong surficial bonding between the SiO_2_ network nano SiO_2_ fibers.

### 2.3. X-Ray Diffraction

**Figure 3:**
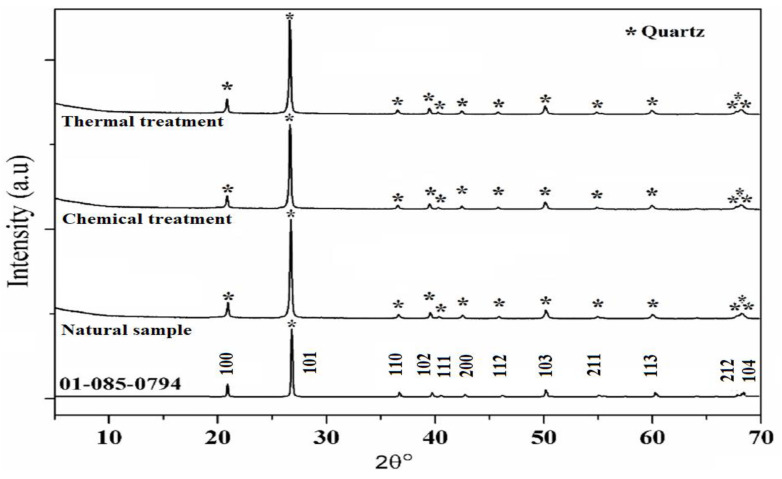
XRD patterns of natural, chemical, and thermal treatments samples.

**Figure 4:**
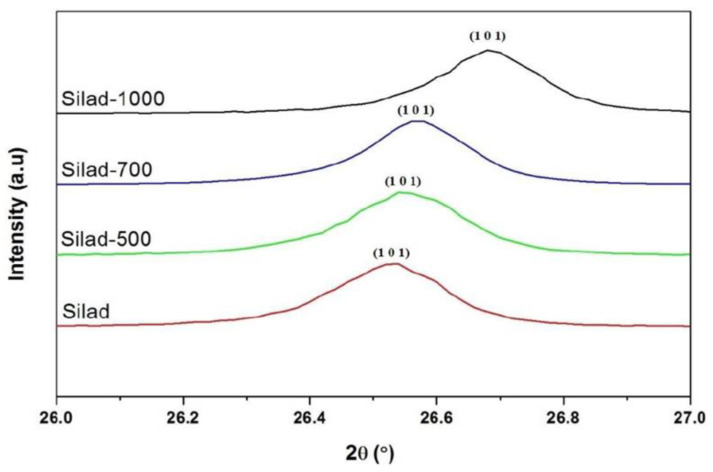
Evolution of the main peak (101) of chemical and thermal treatment samples.

XRD patterns of SiO_2_ samples are shown in Figure 3 in which the aggregate before the treatment (natural sample) is used as a reference. SiO_2_ aggregate has a hexagonal structure related to the SiO_2_ crystalline phase [PDF 01-085-0794] (Figure 4).

In addition, XRD patterns of the samples compared to the reference are presented as well, after the reaction and heat treatment. Peaks corresponding to quartz are present in all samples showing that whatever the treatment, the SiO_2_ lattice is preserved. In fact, the protocol developed in this study does not destroy the initial structure of SiO_2_ (Figure 3). The results suggest that after thermal treatment the XRD peaks are shifted towards higher 2θ values which may be attributed to a contraction of the silica network as is confirmed below (Figure 4).

FWHM of the main peak (101) increases after the chemical reaction which may be attributed to the increase in the SiO_2_ molecular disorder. This molecular disorder generates the phenomenon of dissolution of the siliceous structure leading to the presence of defects in the sample. In addition, chemical stress also causes an increase in the volume of the elementary cell (expansion of the network) as shown in Table 1. This significant volume change (almost 0.6%) can generate variations in internal stresses, as indicated in the following section.

#### Debye–Scherrer Equation and Determination of Crystallite Size

According to the Debye–Scherer method, the crystallite size is inversely proportional to the FWHM of the diffraction peaks. The DD-S crystallite size of SiO_2_ samples is estimated from the following Scherrer equation (Equation (2)):

(2) $$D_{D-S}=\frac{0.9\lambda }{\beta_{hkl}.cos\theta }$$

where D_D−S_ is the crystallite size, λ = 1.5406 Å, and β is FWHM [21]. The results show that crystallite size increases from 63 nm for natural SiO_2_ aggregate to 70 nm after thermal treatment based on the (101) peak. This may be attributed to the improvement of the crystalline quality of SiO_2_.

Different forms of Williamson–Hall methods such as the isotropic strain model, anisotropic strain model, and uniform deformation energy density model were applied in order to investigate the contributions of lattice strain and crystalline size to the XRD peaks. The isotropic strain model (Equation (3)) gives the lattice isotropic strain (ε) and crystallite size (DW–H):

(3) $$\beta .cos\theta =\frac{0.9\lambda }{D_{W-H}}+4\varepsilon sin\theta$$

where β: FWHM, λ: the wavelength of the X-rays, $D_{W-H}$: the crystallite size, ε: the strain, and θ: the diffraction peak angle in degree.

**Figure 5:**
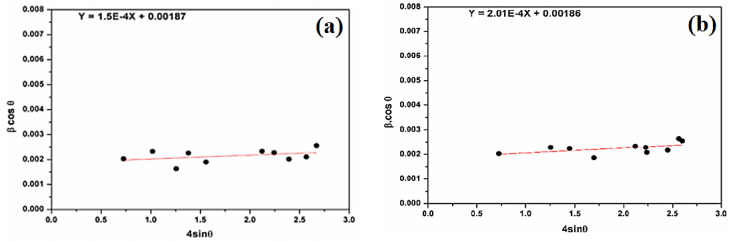
(**a**) β cosθ versus sinθ (W–H plot) for natural SiO_2_ and (**b**) β cosθ versus sinθ (W–H plot) for attacked SiO_2_ samples.

Figure 5 shows (βcosθ) versus (4sinθ) in different samples. The strains (ε) and the crystallite size (DW–H) are deduced from the slope and the βcosθ-intercept of the graph, respectively.

Figure 5 shows the positive deformation of natural SiO_2_ after chemical treatment. The microstrain, estimated at 1.50 × 10^−4^ units for natural SiO_2_, is smaller than after chemical treatment (2.01 × 10^−4^ units). The average crystallites sizes are 74 nm and 75 nm for natural SiO_2_ and SiO_2_ after chemical treatment, respectively. This result is in good agreement with the one calculated by the Scherer formula. In fact, SiO_2_ samples cannot have the same property in all directions.

Hooke’s law is used to describe stress–strain relationships for elastic behavior. Beyond this limit, Hooke’s law is not valid. Another form of Williamson–Hall is given by Equation (4):

(4) $$\beta .cos\theta =\frac{0.9\lambda }{D_{W-H}}+\frac{4\sigma sin\theta }{Y_{hkl}}$$

where σ represents the uniform stress and Yhkl Young’s modulus in the direction perpendicular to the crystal lattice planes (hkl). Young’s modulus in a hexagonal system is calculated using Equation (5) [22]:

(5) $$Y_{hkl}=\frac{[h^{2}+\frac{{\left(h+2k\right)}^{2}}{3}+{\left(\frac{al}{c}\right)}^{2}{]}^{2}}{S_{11}.{\left(h^{2}+\frac{{\left(h+2k\right)}^{2}}{3}\right)}^{2}+S_{33}.{\left(\frac{al}{c}\right)}^{4}+(2S_{13}+S_{44})(h^{2}+\frac{{\left(h+2k\right)}^{2}}{3}){\left(\frac{al}{c}\right)}^{2}}$$

The elastic parameters of the compliance coefficients for SiO_2_ [23] are s11 = 0.01149 GPa^−1^, s33 = 0.00943 GPa^−1^, s13 = 0.08333 GPa^−1^, and s44 = 0.01754 GPa^−1^. Young’s Modulus Yhkl can be determined along any orientation, from the elastic constants (sij).

**Figure 6:**
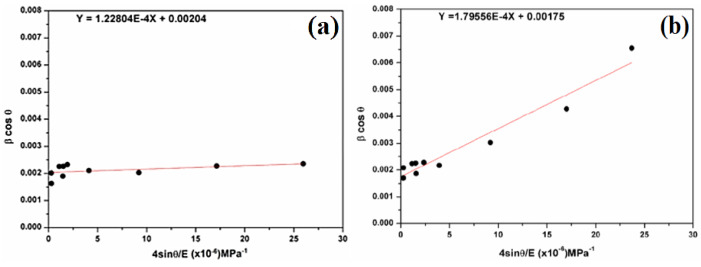
Plots of β cosθ versus 4sinθ/E (**a**) for natural SiO_2_ and (**b**) after chemical treatment.

In Figure 6, the scatter plot is plotted with a regression line of βcosθ versus 4sinθ/E, where (kλ/D_W-H-ASM_) is the βcosθ-intercept with the *y*-axis, and (σ) is the slope of the line. βcosθ tends to increase as sinθ increases, implying a positive correlation. The crystallite size (D_W−H_) is 68 nm for natural SiO_2_ and 79 nm after chemical treatment. In addition, the magnitude of the deformed stress (σ) is found as 122.804 MPa for natural SiO_2_ and 179.56 MPa after chemical treatment.

In order to determine the energy density of a crystal, the introduction of the anisotropic Young’s modulus does not bring significant change in the scattering of data points. In fact, we used another model, such as the uniform deformation energy density model (UDEDM). In addition, to extend the anisotropic approach, Hooke’s law can also be represented as the relation between the strain (ε) and the energy density u (energy per unit volume):

(6) $$u=\frac{\varepsilon^{2}E}{2}$$

According to the energy and strain relation, Equation (3) becomes the following:

(7) $$\beta .cos\theta =\frac{0.9\lambda }{D_{W-H}}+4sin\theta \sqrt{\frac{2u}{E}}$$

**Figure 7:**
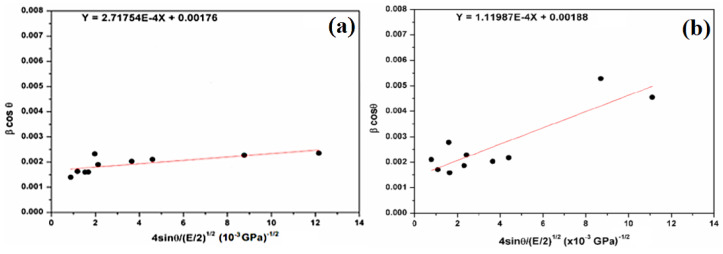
Plots of β cosθ versus 4sinθ/(E/2)^1/2^ (W–H plot) for the natural (**a**) and attacked SiO_2_ samples (**b**).

The regression equation is used to calculate the slope and the intercept (Figure 7). Therefore, the crystallite size (D_W−H_) remains almost the same in comparison with the Scherer method (Equation (2)). The deformation energy density (u) is estimated to be 73.85 kJ/m^3^ and 12.54 kJ/m^3^ for natural SiO_2_ and after chemical treatment, respectively. In our case, all three models give a similar order of crystallite size, which means that the strain has little effect on the crystallite size.

### 2.4. Fourier Transformed Infrared Spectroscopy (FTIR)

**Figure 8:**
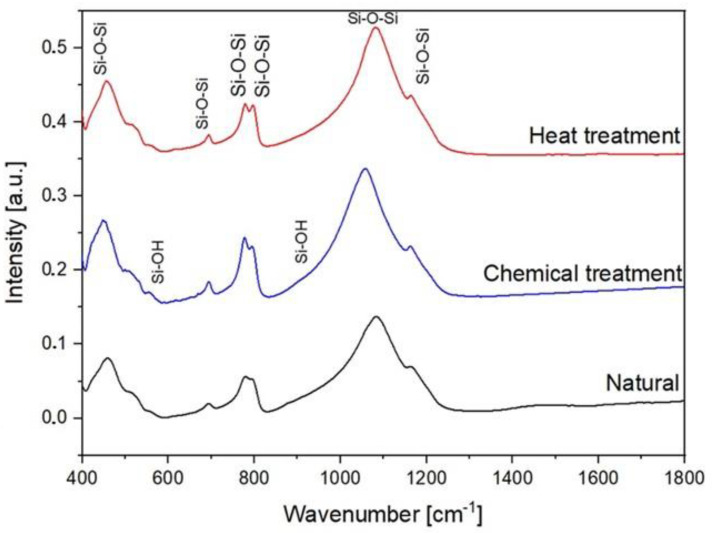
FTIR spectra of natural, chemical, and thermal treatment samples.

In order to investigate various molecular interactions in SiO_2_, FTIR experiments were performed. During FTIR spectroscopy experiments, interatomic bonds (Si–O–Si and Si–O–H as examples) absorb the infrared light of their resonant. The FTIR spectra between 400 cm^−1^ and 1400 cm^−1^ of the natural SiO_2_ samples after chemical and thermal treatment are presented in Figure 8. The IR spectra of all the samples present a broadband between 1000 cm^−1^ and 1300 cm^−1^. This band consists of two strong peaks located at 1078 cm^−1^ and 1163 cm^−1^. These peaks are attributed to the stretching vibration of Si–O–Si [24,25]. In addition, the bending vibrations attributed to Si–O–Si [26,27] located at 455, 509, 778, and 800 cm^−1^ are observed. The bands located at 555 cm^−1^ and 950 cm^−1^ are attributed to the Si-O bending vibrations of non-bridging Si–OH bonds and the stretching vibration, respectively [28,29].

Non-bridging Si–O bonds are formed when H+ occupies the residual charge of non-bridging Si–O−, forming Si–OH. Thus, the resulting Si–O vibration is expected to have a higher natural frequency than that of the Si–O vibrations in bridging Si–O–Si. The non-bridging Si–O bending vibration can also be expected in hydroxylated SiO_2_ glass-like opal-A where a part of the Si–O–Si bonds is interrupted by the incorporation of hydrogen protons [30].

**Figure 9:**
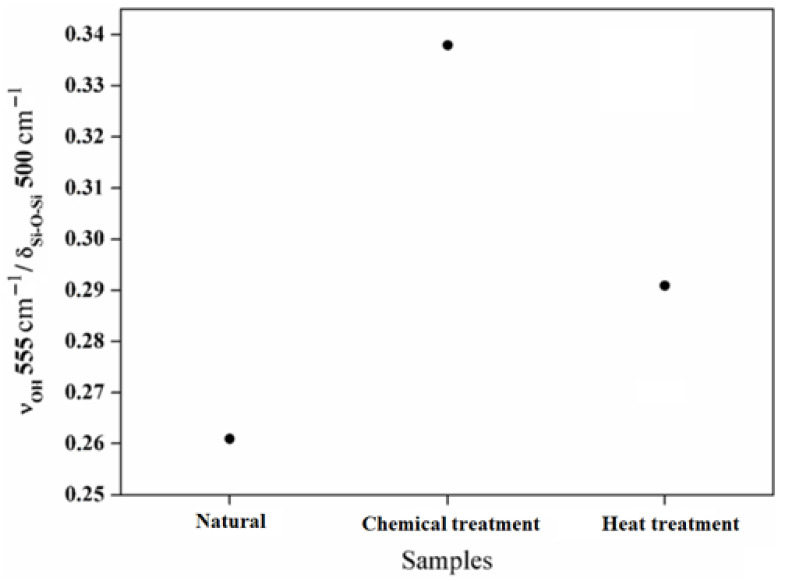
The intensity ratio between the Si-OH bands located around 555 cm^−1^ and the structural band located around 500 cm^−1^.

In Figure 9, the intensity ratio between the Si–OH groups around 555 cm^−1^ and the structural bands around 500 cm^−1^ are shown. The intensity ratio between the Si–OH groups and the Si–O–Si bands increases with the chemical reaction and decreases with the heat treatment. This evolution demonstrates the evaporation of the hydroxyl groups Si–OH. Based on these results, it is possible to have control of the surface behavior of the aggregates chemically and thermally. The deformations of the atomic network inferred via micromechanical constraints are closely linked to the molecular changes inferred by infrared spectroscopy. These changes are also observed from the nanomorphology thanks to high-resolution transmission electron microscopy. The combination of these results added to new investigations will allow us to draw a model linking micro- and macromechanical properties.

## 3. Materials and Methods

### 3.1. Sample Preparation

**Figure 10:**
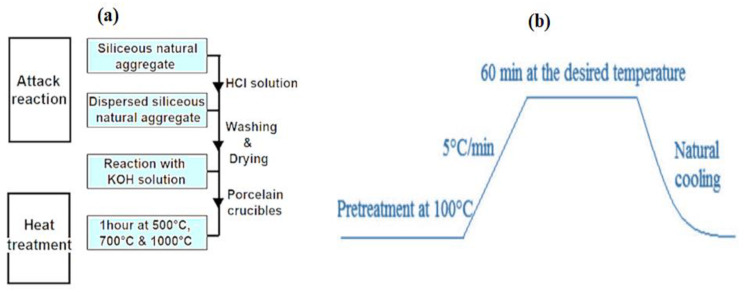
Preparation protocol diagram: (**a**) chemical attack and heat treatment and (**b**) heat treatment procedure of the samples.

Figure 10 summarizes the sample processing protocol, whereby 1g of silica aggregate is introduced into an autoclave and placed in an oven at 80 °C for 30 min. After this first step of heating, 10 mL of KOH solution at 0.79 mol/L is added (to have a basic environment pH = 13.9). Then, the autoclave is placed in an oven under a controlled temperature and reaction time. In order to stop the reaction, the autoclave is put in a bath of frozen water for 5 min. Using an acid treatment of 0.5 M HCl solution, the soluble reaction products are removed. The silica is then recovered by vacuum filtration on a millipore paper filter and then chemically dried by successive rinses with distilled water, acetone, and ether. To avoid their carbonation and hydration (Figure 10a), the samples obtained are stored in a desiccant.

In addition, heat treatment was carried out under the following conditions:

The obtained samples were submitted to thermal treatment (500 °C, 700 °C, and 1000 °C) in a porcelain crucible under the heat treatment procedure as shown in Figure 10b below.

### 3.2. Characterization

Various techniques were used to characterize the reacted and heated samples such as X-ray diffraction (XRD), variable pressure scanning electron microscopy (VP-SEM), HR transmission electron microscopy (HR-TEM), Fourier transformed infrared spectroscopy (FTIR), and thermogravimetry analysis (TGA). In addition, in order to check the purity of the SiO_2_ aggregate after chemical treatment, energy-dispersive spectrometry (EDS) was used.

#### 3.2.1. VP-SEM and HR-TEM

To observe the morphology of the SiO_2_ aggregates at different steps of the treatment, the VEGA 3 instrument model product by TESCAN (Brno, Czech Republic) equipped with an energy-dispersive X-ray spectrometer (EDS) was used. The instrument is a VP-SEM operating at a low energy of 10 keV, in order to avoid the degradation of the samples which are observed without any coating. In addition, in order to observe the morphology of the SiO_2_ surface, a HR-TEM Titan Themis STEM model (Thermo Fisher Scientific, Waltham, MA, USA) (80 kV, for operating at low energy keeps the sample free from electron beam artifacts) equipped with an EDS spectrometer.

#### 3.2.2. X-Ray Diffraction

Using a Bruker D8 Advance diffractometer (Bruker, Billerica, MA, USA) operating at 40 kV and 40 mA (λCu-K = 1.5418 A°), X-ray diffraction patterns were collected in reflection mode. The intensity of diffraction versus 2θ° was plotted. Data were recorded in the range of 25°–75° (in the 2θ scale) with a step size of 0.02° and 0.5 s/step. HighScore 5.1 software was used for data treatment.

#### 3.2.3. Fourier Transformed Infrared Spectroscopy (FTIR)

A Brucker VERTEX 70 spectrometer (Bruker, Billerica, MA, USA) in reflection mode was used to acquire the FTIR spectra of SiO_2_ aggregates at different steps. Each spectrum was collected after 100 scans with a resolution of 4 cm^−1^ in the range of 400–4000 cm^−1^.

## 4. Conclusions

The micromechanical, chemical, and thermal properties of SiO_2_ flint aggregate were investigated. Using different techniques such as VP-SEM, XRD, FTIR, TDA, TGA, and HR-TEM, new SiO_2_ samples provide a promising functionalized surface with new silica nanofiber crystallinity and different chemical and thermal treatments. The presence of SiO_2_ nanofibers on the surface is related to the microstrain, estimated at 1.50 × 10^−4^ units for natural SiO_2_ and 2.01 × 10^−4^ units after chemical treatment.

Our investigation established that SiO_2_ characterized with controlled micromechanical, thermal, and chemical properties may be used as a filler to improve all the performance properties of the strength, microstructure, and durability of some composites. The use of controlled surface SiO_2_ as filler is beneficial for its environmental aspect and used in new applications.

## Figures and Tables

**Table 1:** The microstructure parameters of SiO_2_ Samples.

Samples		Structure	FWHM (101)	Lattice Parameter		
				a = b (Ǻ)	c (Ǻ)	Lattice Volume (Ǻ^3^)
Natural sample		Hexagonal	0.1968	4.92937 ± 0.00009	5.47631 ± 0.00018	115.1829 ± 0.0048
Chemical treatment			0.1574	4.89835 ± 0.00009	5.38702 ± 0.00018	111.9401 ± 0.0048
Thermal treatments	500 °C		0.1771	4.69494 ± 0.00015	5.47950 ± 0.00028	104.6002 ± 0.0065
	700 °C		0.1474	4.69165 ± 0.00014	5.47826 ± 0.00028	104.4297 ± 0.0065
	1000 °C		0.1378	4.42348 ± 0.00013	4.95383 ± 0.00023	83.9459 ± 0.0054

## Data Availability

Analytical data will be provided upon reasonable request to the corresponding author.
